# Hydrogen Oxidation Influences Glycogen Accumulation in a Verrucomicrobial Methanotroph

**DOI:** 10.3389/fmicb.2019.01873

**Published:** 2019-08-16

**Authors:** Carlo R. Carere, Ben McDonald, Hanna A. Peach, Chris Greening, Daniel J. Gapes, Christophe Collet, Matthew B. Stott

**Affiliations:** ^1^Department of Chemical and Process Engineering, University of Canterbury, Christchurch, New Zealand; ^2^Scion, Te Papa Tipu Innovation Park, Rotorua, New Zealand; ^3^Geomicrobiology Research Group, Department of Geothermal Sciences, GNS Science, Taupō, New Zealand; ^4^School of Biological Sciences, Monash University, Clayton, VIC, Australia; ^5^School of Biological Sciences, University of Canterbury, Christchurch, New Zealand

**Keywords:** methanotroph, hydrogenase, glycogen, extremophile, methylacidiphilum

## Abstract

Metabolic flexibility in aerobic methane oxidizing bacteria (methanotrophs) enhances cell growth and survival in instances where resources are variable or limiting. Examples include the production of intracellular compounds (such as glycogen or polyhydroxyalkanoates) in response to unbalanced growth conditions and the use of some energy substrates, besides methane, when available. Indeed, recent studies show that verrucomicrobial methanotrophs can grow mixotrophically through oxidation of hydrogen and methane gases *via* respiratory membrane-bound group 1d [NiFe] hydrogenases and methane monooxygenases, respectively. Hydrogen metabolism is particularly important for adaptation to methane and oxygen limitation, suggesting this metabolic flexibility may confer growth and survival advantages. In this work, we provide evidence that, in adopting a mixotrophic growth strategy, the thermoacidophilic methanotroph, *Methylacidiphilum* sp. RTK17.1 changes its growth rate, biomass yields and the production of intracellular glycogen reservoirs. Under nitrogen-fixing conditions, removal of hydrogen from the feed-gas resulted in a 14% reduction in observed growth rates and a 144% increase in cellular glycogen content. Concomitant with increases in glycogen content, the total protein content of biomass decreased following the removal of hydrogen. Transcriptome analysis of *Methylacidiphilum* sp. RTK17.1 revealed a 3.5-fold upregulation of the Group 1d [NiFe] hydrogenase in response to oxygen limitation and a 4-fold upregulation of nitrogenase encoding genes (*nifHDKENX*) in response to nitrogen limitation. Genes associated with glycogen synthesis and degradation were expressed constitutively and did not display evidence of transcriptional regulation. Collectively these data further challenge the belief that hydrogen metabolism in methanotrophic bacteria is primarily associated with energy conservation during nitrogen fixation and suggests its utilization provides a competitive growth advantage within hypoxic habitats.

## Introduction

Aerobic methane oxidizing bacteria (methanotrophs) serve as the primary biological sink for the potent greenhouse gas methane (CH_4_) (Kirschke et al., 2013). Methanotrophs grow by oxidizing CH_4_ to methanol with a particulate or soluble methane monooxygenase enzyme (pMMO/sMMO) and subsequently yield reducing equivalents (e.g., NADH) for cellular respiration and biosynthesis through the oxidation of methanol to carbon dioxide (CO_2_). The gammaproteobacterial (Type I) and alphaproteobacterial (Type II) methanotrophs generate biomass by assimilating the intermediates formaldehyde or formate via the ribulose monophosphate (RuMp) or serine pathways (Hanson and Hanson, 1996) respectively, whereas the verrucomicrobial methanotrophs oxidize methanol directly to formate (Keltjens et al., 2014) and generate biomass by fixing inorganic carbon (CO_2_) via the Calvin–Benson–Bassham cycle (Khadem et al., 2012b). Despite the apparent restriction of most methanotrophs to grow on one carbon compounds (C1), they thrive at the interface of various oxic/anoxic habitats (e.g., peat bogs, forest soils, wetlands, rice paddies and geothermal environments) (Dunfield et al., 2007; Singh et al., 2010; Knief, 2015), where the availability of oxidant (O_2_), energy and carbon resources for growth is likely to fluctuate. Given the methane monooxygenase reaction (CH_4_ + O_2_ + [NAD(P)H + H^+^]/QH_2_ CH_3_OH + NAD(P)^+^/Q + H_2_O) and the aerobic respiratory chain require a continual source of reductant and oxidant, methanotrophic bacteria must regulate their carbon, energy and resource allocation to fulfill metabolic demands for cellular growth and persistence (Hanson and Hanson, 1996).

Many bacterial species, including methanotrophs, accumulate biopolymers (e.g., glycogen, polyhydroxyalkanoates), phospholipids, and intracellular osmolytes (e.g., ectoine, sucrose) (Strong et al., 2016) in response to unbalanced growth conditions. This allows resources to be strategically conserved for assistance in times of starvation. The biosynthesis of glycogen, a highly branched polysaccharide consisting of α-1,4 bonded glucose residues with additional α-1,6 branched sidechains, is a common metabolic strategy for carbon storage that is shared among evolutionarily distant species (Wilson et al., 2010). Glycogen production has been widely described within Type I methanotroph species (Linton and Cripps, 1978; Eshinimaev et al., 2002) and the production of this compound has recently been reported in the verrucomicrobial methanotroph, *Methylacidiphilum fumarolicum* SolV (Khadem et al., 2012a). The physiological role of glycogen production in methanotrophs is not precisely understood, although it is believed to serve a role in environmental survival during periods of starvation and has been implicated to symbiotic performance, colonization and virulence (Bonafonte et al., 2000; McMeechan et al., 2005; Bourassa and Camilli, 2009; Wilson et al., 2010). Although the accumulation of intracellular glycogen may occur optimally during exponential growth (Gibbons and Kapsimalis, 1963; Eidels and Preiss, 1970), its synthesis is typically associated with entry into stationary phase when growth is limited due to the limitation of some critical nutrient (i.e., nitrogen, phosphate) or in the presence of excess carbon (Wilson et al., 2010). In bacteria, the biosynthesis of glycogen occurs by utilizing ADP-glucose as the glycosyl donor for polymer extension (Preiss, 1984). The precise mechanisms governing glycogen biosynthesis in bacteria, however, remain obscure. It is likely energy availability and redox status play a primary role in regulating glycogen biosynthesis, as ATP acts as substrate for the ADP-glucose producing reaction catalyzed by glucose-1-phosphate adenylyltransferase (Preiss, 1984).

To remain competitive within dynamic environments (Knief et al., 2003; Tavormina et al., 2010), some methanotrophs supplement CH_4_ usage with other energy-yielding strategies (Dedysh and Dunfield, 2010). Several recent studies have revealed a few strains, notably *Methylocella silvestris*, utilize a suite of carbon and energy substrates, including simple organic acids, alcohols and short-chain alkane gases (Dedysh et al., 2005; Crombie and Murrell, 2014). Aerobic H_2_ metabolism has also been shown in a range of methanotrophs (Chen and Yoch, 1987; Shah et al., 1995; Hanczar et al., 2002) and a wide range of hydrogenases have been shown to be distributed in methanotroph genomes (Greening et al., 2016). While H_2_ oxidation was originally implicated in energy conservation in response to N_2_ fixation (Takeda, 1988), more recent findings indicate that H_2_ serves a multifaceted role in the growth and survival of these bacteria. Of the verrucomicrobial methanotrophs, the activity of respiratory-linked group 1d hydrogenases can provide sufficient energy to sustain chemolithoautotrophic growth on H_2_ alone (Mohammadi et al., 2016; Carere et al., 2017). Further, mixotrophic growth (H_2_ and CH_4_) in the thermoacidophile *Methylacidiphilum* sp. RTK17.1 has been observed under O_2_-limiting conditions and is proposed to provide a competitive advantage over obligate methanotrophy at oxic/anoxic soil boundaries within geothermal environments (Carere et al., 2017). This suggests that the additional energetic input of H_2_ may counter the effect of otherwise unbalanced growth conditions.

The influence of H_2_ metabolism on the production of intracellular energy reservoirs, commonly associated with unbalanced growth, within methanotrophic bacteria has yet to be elucidated. In this work, we investigate the effect of H_2_ metabolism on glycogen production within the methanotroph, *Methylacidiphilum* sp. RTK17.1. Chemostat cultivation was performed during O_2_-replete and O_2_-limited cultivation, in the presence of NH_4_^+^ or N_2_, with or without H_2_ in the headspace, to determine the influence of H_2_ metabolism on observed growth rates, biomass production characteristics and transcriptional regulation. We show that cellular growth rates, molar growth yields, and the allocation of resources between protein and glycogen production vary depending on the supply of H_2_, O_2_, and nitrogen (as NH_4_^+^ or N_2_). Transcriptome data provided a basis of findings, showing significant differential regulation of operons encoding the group 1d [NiFe]-hydrogenase, methane monooxygenases, and nitrogenase between the conditions. In turn, these findings enhance understanding of the physiological strategies that methanotrophs use to grow and survive in different environments.

## Materials and Methods

### Chemostat Cultivation of *Methylacidiphilum* sp. RTK17.1

Chemostat cultivation was performed to investigate the influence of H_2_ metabolism on the growth and production of intracellular glycogen reservoirs in *Methylacidiphilum* sp. RTK17.1 with respect to unbalanced growth conditions (O_2_ and nitrogen limitation). As previously described, a 1 l bioreactor (BioFlo 110; New Brunswick Scientific, Edison, NJ, United States) equipped with an InPro 6810 Polarographic Oxygen Sensor (Mettler-Toledo, Columbus, OH, United States) was used for all cultivations (Carere et al., 2017). Cultures were continuously incubated at pH 2.5, 50°C with stirring (800 rpm). The reactor was constantly maintained at a volume of 0.5 l with V4 mineral medium (Carere et al., 2017), prepared with or without NH_4_Cl (0.4 g l^–1^) addition (as necessary), and supplied at a constant flow rate of 10 ml h^–1^ (*D* = 0.02 h^–1^). Custom gas mixtures were supplied to the chemostat at a rate of 10 ml min^–1^ and contained approximately (v/v) 3% CH_4_ and 26% CO_2_ for all experiments; O_2_ at (v/v) 14.1% and 3.5%, respectively for O_2_-replete and -limiting conditions, and H_2_ at 0.4% (v/v). The balance of all gas mixtures was made up with N_2_.

Cell densities were monitored at 600 nm using a Ultrospec 10 cell density meter (Amersham Bioscience, United Kingdom) with one unit of OD_600_ equivalent to 0.43 g l^–1^ cell dry weight for *Methylacidiphilum* sp. RTK17.1. Influent and effluent gas concentrations were monitored using a 490 micro GC equipped with a thermal conductivity detector (Agilent Technologies, United States). After achieving a steady-state condition as determined by OD_600_, gas concentrations were monitored over several days and used as a basis to calculate growth and specific gas consumption rates. Biomass samples of *Methylacidiphilum* sp. RTK17.1 were harvested during steady-state operation for subsequent transcriptome sequencing, biomass cell dry weight determinations, intracellular glycogen, total protein and amino acid levels measurements.

### Transcriptome Sequencing

Cell culture samples for transcriptome sequencing were harvested (10 ml) from steady-state chemostat experiments, pelleted by centrifugation at 5,000 x g (15 min, 4°C), suspended in 1 ml RNAlater Stabilization solution (Thermo Fisher Scientific) and then stored at −80°C until required for further analysis, as per the manufacturer’s recommended protocols. The extraction and sequencing of RNA was performed by Macrogen Inc. (Seoul, Korea). Briefly, isolation of mRNA was performed using the RNeasy Mini kit (Qiagen) according to the manufacturer’s protocol. Following total RNA extraction, ribosomal RNAs were removed using the Ribo-Zero rRNA removal kit (bacteria) and the quality of the remaining RNA was assessed using an Agilent 2100 Bioanalyzer (Agilent). Library construction was performed using the TruSeq Stranded Total RNA Sample Prep (microbe) Kit (Illumina) and sequencing was performed using an Illumina HiSeq2500 platform. From this, an average of 7,988,451 raw untrimmed reads were obtained for each of the five conditions sampled. These reads were then analyzed using the Artificial Intelligence RNA-Seq pipeline (Sequentia Biotech, Barcelona, Spain), as described elsewhere (Vara et al., 2019), which were reduced to an average of 7,287,318 following quality filtering and trimming. Retained paired-end reads (100 bp) were then mapped to the genome of *Methylacidiphilum infernorum* strain V4 (GCA_000019665.1) (Hou et al., 2008) using the ‘different genotype’ setting. An average of 80.01% reads were mapped to genes within the reference genome for the five experimental conditions (condition 1: O_2_ limiting, N_2_, no H_2_ addition; condition 2: O_2_ limiting, N_2_, H_2_ addition; condition 3: O_2_ limiting, NH_4_^+^, H_2_ addition; condition 4: O_2_ replete, NH_4_^+^, H_2_ addition; condition 5: O_2_ replete, NH_4_^+^, no H_2_ addition). Following this, differential gene expression profiles and accompanying statistical analysis was performed to investigate regulation using the edgeR (Robinson et al., 2010) tool available within the Artificial Intelligence RNA-Seq pipeline. Synonymous conditions were grouped as replicates for differential gene expression analysis during oxygen limiting (conditions 1, 2, and 3) and oxygen excess (conditions 4 and 5) growth. Likewise, conditions were grouped as replicates for differential gene expression analysis under nitrogen fixing (N_2_; conditions 1 and 2) and nitrogen excess (NH_4_^+^; conditions 3, 4, and 5) growth conditions, respectively. Where provided, expression values are given as FPKM (Fragments per Kilobase Million; Supplementary Table S1) (Mortazavi et al., 2008). Raw and processed transcriptome sequence files (accession numbers GSM3872525-GSM3872529) were subsequently deposited into the Gene Expression Omnibus (GEO^1^) for archival storage.

### Characterization of Biomass

Effluent biomass, produced during experimental steady-state chemostat operation, was collected and stored at 4°C over a period of 7 days for all biomass characterization studies. Following collection of approximately 2 l culture, cells were pelleted (5,000 × *g*, 20 min, 4°C) and stored at −20°C until required. Characterization of *Methylacidiphilum* sp. RTK17.1 biomass (crude protein, ash content, amino acid composition) was performed at the Massey University Nutrition Laboratory (accredited to ISO 17025; New Zealand) according to the official methods of analysis of the Association of Official Analytical Communities (AOAC, 2005) international. Briefly, total crude protein and ash content (% w/w) were determined via the Dumas method (AOAC method 968.06) (Ebeling, 1968) and furnace methods, respectively (AOAC method 942.05) (Thiex et al., 2012). Amino acid profile determination of acid-stable residues was performed via reverse-phase high performance liquid chromatography (HPLC) separation using AccQ derivatization of biomass (60–140 mg) samples following oxidization with performic acid and hydrolysis with hydrochloric acid as described in AOAC method 994.12 (AOAC, 2005).

The concentration of glycogen within crude cell extracts of *Methylacidiphilum* sp. RTK17.1 was determined using a Dionex ICS 3000 HPLC equipped with a Biorad Aminex HPX-87H column and a Shodex RI-101 refractive index detector. Triplicate cell pellets, dried to constant weight (5–15 mg), were suspended in 1M NaOH (0.9 ml) and disrupted by boiling lysis for 1 h within sealed screw top Micro Tubes (Thermo Fisher Scientific). The efficacy (>99%) of cell lysis was confirmed microscopically. Crude extracts were then incubated with amyloglucosidase (35 U/ml crude) from *Aspergillus niger* (Sigma-Aldrich), following acidification with acetate buffer (0.1 ml) to pH 4.8, for 8 h at 45°C to convert intracellular glycogen reservoirs into glucose. Following this, the resulting glucose was quantified by HPLC and normalized against a standard curve of known glycogen content (Sigma) that had been contemporaneously treated with amyloglucosidase. To account for non-glycogen derived glucose in cell samples, extracts that did not undergo amyloglucosidase treatment were similarly analyzed by HPLC in parallel. Total intracellular glycogen content was then expressed as a function of cell dry weight (% w/w). Observed differences in growth and biomass characteristics were analyzed for statistical significance using the Tukey’s honest significance test tool available in Prism v7.0a (Graphpad Software, Inc.).

## Results

### Hydrogen Availability Differentially Affects Growth and Biomass Allocation Depending on Oxygen and Nitrogen Availability

The growth characteristics and biomass production of *Methylacidiphilum* sp. RTK17.1 was compared in chemostats in four different conditions that differed in O_2_, nitrogen (NH_4_^+^/N_2_), and H_2_ supply. Results from these experiments are provided in Table 1.

**TABLE 1:** Growth and productivity characteristics of *Methylacidiphilum* sp. RTK17.1 during chemostat cultivation.

**Growth condition^a,b,c^**	**O_2_-limited, N_2_ no H_2_ addition**	**O_2_-limited, N_2_ H_2_ addition**	**O_2_-limited, NH_4_^+^ H_2_ addition**	**O_2_-replete, NH_4_^+^ H_2_ addition**
**Biomass productivity:**				
mg l^–1^ h^–1^	4.33 (± 0.10)	5.09 (± 0.19)	5.57 (± 0.50)	8.32 (± 0.10)
CH_4_ consumption rate:				
mmol l^–1^h^–1^	0.76 (± 0.05)	0.69 (± 0.06)	0.89 (± 0.05)	1.28 (± 0.10)
mmol gCDW^–1^ h^–1^	3.31 (± 0.21)	2.73 (± 0.27)	3.18 (± 0.13)	3.22 (± 0.05)
Y_CDW/CH__4_ (g mol^–1^)	5.73 (± 0.35)	7.39 (± 0.74)	6.29 (± 0.25)	6.52 (± 0.11)
H_2_ consumption rate:				
mmol l^–1^ h^–1^	_–_	0.19 (± 0.01)	0.20 (± 0.01)	0.01 (± 0.01)
mmol gCDW^–1^ h^–1^	–	0.71 (± 0.10)	0.71 (± 0.08)	0.02 (± 0.01)
**Glycogen content:**				
% CDW	48.86 (± 4.32)	20.00 (± 2.93)	20.23 (± 0.77)	11.26 (± 0.14)
mg glycogen l^–1^h^–1^	2.12 (± 0.19)	1.04 (± 0.15)	1.13 (± 0.04)	0.94 (± 0.01)

*^*a*^Feedgas was continuously supplied at a rate of 10 ml min*^–^*^1^ with the following compositions (where applicable;% v/v): O_2_-replete, 14.1%; O_2_-limiting, 3.5%, H_2_ 0.4%. For all experiments excess CH_4_ was supplied at 3.2%, CO_2_ at 26% with the balance made up with N_2_. O_2_ saturations in the medium were 57.5 and 0.17% for the O_2_-replete and O_2_-limiting conditions, respectively. ^*b*^A dilution rate of 0.02 h^–1^ (10 ml h^–1^) was maintained for all experiments, with NH_4_^+^ supplied at an influent concentration of 0.4 g l^–1^ where applicable. ^*c*^Replicate numbers for each growth condition correspond to *n* = 6, *n* = 6, *n* = 4 and *n* = 7 moving from the leftmost column to rightmost column, respectively. The standard deviation from the average of these measurements is shown in brackets.*

Under nitrogen-replete conditions (i.e., NH_4_^+^ present in the medium), *Methylacidiphilum* sp. RTK17.1 displayed many of the same growth characteristics in response to O_2_ availability as found previously (Carere et al., 2017) and results were consistent with similar studies on *M. fumarolicum* SolV (Khadem et al., 2012a). The observed rate of steady-state biomass production (mg l^–1^h^–1^) of *Methylacidiphilum* sp. RTK17.1) decreased by 33.1% following the transition from O_2_-replete to O_2_-limiting cultivation (*p-value*: <0.0001). This decrease in growth rate was accompanied by a 30.4% reduction in the volumetric rate (mmol l^–1^ h^–1^) of CH_4_ consumption and a 19-fold increase in the observed rate of H_2_ oxidation (*p-value*: <0.0001). With respect to specific gas consumption rates normalized against biomass production (mmol gCDW^–1^ h^–1^), observed rates of CH_4_ consumption did not change significantly (*p-value*: 0.988), whereas H_2_ consumption increased 36-fold (*p-value*: <0.0001). Intracellular glycogen content was observed to increase dramatically from 11.26 (± 0.14)% during O_2_-replete growth to 20.23 (± 0.77)% (w/w; *p-value*: < 0.0001) during O_2_-limiting cultivation.

We observed significant changes in these parameters when *Methylacidiphilum* sp. RTK17.1 was cultivated under O_2_-limitation depending on H_2_ and nitrogen supply. In the presence of H_2_, observed rates of *Methylacidiphilum* sp. RTK17.1 biomass production decreased by 8.61% (*p-value*: 0.019) during N_2_-fixation compared to growth on NH_4_^+^ replete media. In comparison, removal of H_2_ from the headspace resulted in an 18.67% reduction in the observed rate of biomass production compared to cells cultured in NH_4_^+^-replete media (from 5.57 to 4.33 mg l^–1^ h^–1^; Table 1; *p-value*: < 0.0001). These results suggest the activity of respiratory-linked aerobic H_2_ oxidation may, at least partially, serve to offset the energetic demand imposed by N_2_ fixation. In support of this, onset of nitrogen limitation in cultures actively respiring H_2_ did not significantly increase the production of intracellular glycogen (*p-value*: 0.999). Following the removal of H_2_ from the reactor feedgas, however, internal glycogen content significantly increased from 20.00 (± 2.93)% to 48.86 (± 4.32)% (w/w; *p-value*: < 0.0001). This corresponds to a 2.03 fold increase in the volumetric production rate of glycogen (Table 1). In the absence of CH_4_, at 4°C, cellular glycogen reservoirs were depleted within 100 days (Supplementary Figure S1).

**FIGURE 1:**
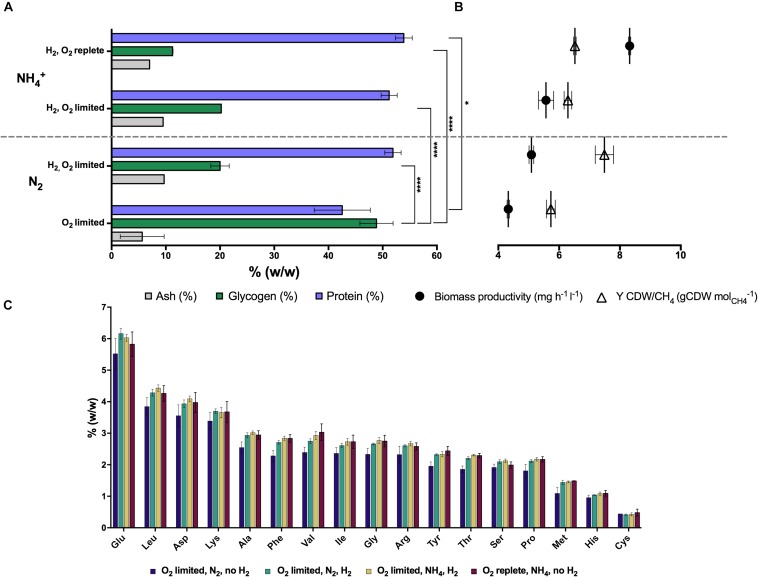
The production of intracellular glycogen in chemostat grown cultures of *Methylacidiphilum* sp. RTK17.1 is influenced by O_2_ availability, and H_2_ and nitrogen metabolism. **(A)** Total protein, ash and glycogen content of cells, **(B)** observed growth rates and biomass yields (CDW: cell dry weight), and **(C)** amino acid content profiles are shown relative to total biomass. For all chemostat growth conditions, *Methylacidiphilum* sp. RTK17.1 excess CH_4_ was continuously supplied (3% v/v, 10 ml min^– 1^). Displayed values represent the average of minimum triplicate samples, with error bars illustrating the standard deviation. Significant differences in cellular glycogen and protein content are shown next to squared brackets (^****^p-value < 0.0001, ^*^p-value < 0.05).

The total protein and amino acid content of *Methylacidiphilum* sp. RTK17.1 biomass produced during steady-state growth was also determined (Figure 1). A maximum total protein content of 53.9 (± 2.69)% (w/w) was achieved during growth under O_2_-replete conditions with NH_4_^+^ as a readily available source of nitrogen. Compared to O_2_-replete growth, a minor but insignificant decrease in total protein content of biomass coincided with reduced O_2_ availability (51.2 ± 2.56%), and in the transition from NH_4_^+^ to N_2_ as a source of nitrogen (51.9 ± 2.56%). Consistent with the observation that glycogen production increased in the absence of H_2_ (under O_2_-limiting, N_2_-fixing growth conditions), the least total protein content of biomass (42.5 ± 8.94%) was observed in conditions where energy availability is likely to constrain cell growth (i.e., O_2_-limiting, N_2_-fixing, no H_2_; *p-value*: 0.018; Figure 1A). Observed changes in the concentration of specific amino acid residues under all of the growth conditions were generally consistent with the changes associated with total protein determinations (Figure 1C). For each of the experimental conditions, glutamic acid, leucine, aspartic acid, lysine and alanine were the amino acids in greatest abundance whereas methionine, histidine and cysteine were the least abundant residues.

### Transcriptome Analysis Reveals Changes in Hydrogenase, Nitrogenase, and Methane Monooxygenase Expression Between the Conditions

**FIGURE 2:**
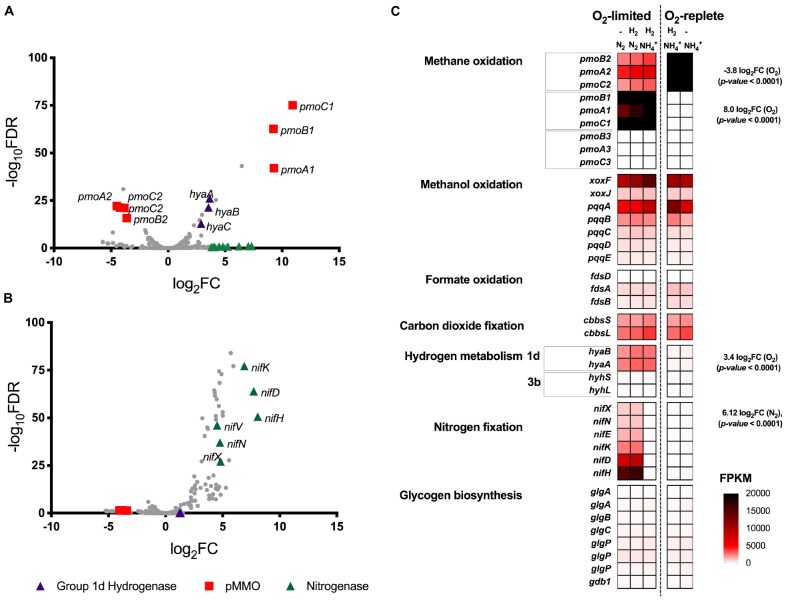
Differential gene expression profiles of chemostat-grown cultures of *Methylacidiphilum* sp. RTK17.1 grown under O_2_-replete and O_2_-limiting conditions in the presence of ammonium (NH_4_^+^) or under N_2_-fixing conditions. **(A)** Volcano plot showing differential gene expression changes following the transition from oxygen replete to O_2_-limited growth. **(B)** Volcano plot showing differential gene expression changes following the transition from nitrogen-replete (NH_4_^+^) to N_2_-fixing growth conditions. Both volcano plots compare data generated from the same five transcriptomes with fold-change values (log_2_FC) and false discovery rates (FDR) calculated using O_2_-replete and nitrogen excess as reference conditions, respectively. Each gene is represented by a gray dot and genes of interest are highlighted as per the legend. **(C)** Heat map of transcript abundance for key genes encoding the structural subunits of enzymes participating in methane oxidation (*pmoBAC;* particulate methane monooxygenase), methanol oxidation (*xoxFJ;* methanol dehydrogenase, *pqqABCDE;* pyrroloquinoline biosynthesis), formate oxidation (*fdsDAB*; formate dehydrogenase), carbon-dioxide fixation (*cbbsSL*; Rubisco), hydrogen metabolism (*hyaBA and hyhSL;* encoding group 1d and 3b [NiFe]-hydrogenases respectively), nitrogen fixation (*nifXNEKDH; nitrogenase*), and glycogen biosynthesis (*glgAABCPPP1*). The fragment counts per kilobase million transcripts (FPKM) are shown for steady-state cultures. O_2_-replete, O_2_-limiting, nitrogen-replete (NH_4_^+^), N_2_-fixing and the supplementation of H_2_ into the feed gas during chemostat operation is indicated.

Transcriptome analysis was performed on chemostat grown cultures of *Methylacidiphilum* sp. RTK17.1 to determine whether genes associated with energy metabolism (CH_4_ or H_2_), glycogen synthesis, N_2_ or CO_2_ fixation were regulated in response to O_2_ or nitrogen availability (Figure 2). For these experiments, to resolve the possible influence of H_2_ on transcriptional responses, a fifth chemostat condition (condition 5: O_2_ replete, NH_4_^+^, no H_2_ addition) was added to the four growth conditions described in Table 1. In response to O_2_ limitation, 36 genes were identified as significantly upregulated (*p-value*: <0.001; False discovery rate (FDR): < 0.05) and 36 genes were significantly downregulated (*p-value*: < 0.001; FDR: < 0.05). Subunits of the particulate methane monooxygenase operon (*pmoCAB1*), corresponding to *M. infernorum* V4 loci Minf_1509–1511, displayed the greatest degree of transcriptional upregulation (average: 9.8 Log_2_FC, *p-value*: < 0.0001) in response to O_2_ limitation. In contrast, the closely related and immediately proximal *pmoCAB2* operon (homologous to *M. infernorum* V4 loci Minf_1506–1508) that was highly expressed during O_2_ replete growth, was strongly downregulated during O_2_-limited growth (average: −4.0 Log_2_FC, *p-value*: < 0.0001). Interestingly, transcripts for a third putative and relatively divergent *pmoCAB3* operon were not detected under the experimental conditions tested.

Expression of the complete complement of genes necessary for the oxidation of CH_4_ to CO_2_, carbon assimilation via the Calvin-Benson-Bassham cycle, and ATP production via aerobic respiration were detected in all conditions tested (Figure 2C and Supplementary Table S1). However, with exception of a 1.78-fold (*p-value*: < 0.0001) downregulation of a *moxY*-like gene (Minf_1448) that encodes a methanol utilization control sensor protein, no significant differences in expression level in these pathways were observed across conditions. With respect to glycogen metabolism, all genes necessary for glycogen synthesis, storage and degradation were expressed, but did not display evidence of transcriptional regulation (Supplementary Table S1). Consistent with the onset of H_2_ oxidation in chemostat cultures, genes encoding for the large and small subunits of the Group 1d respiratory [NiFe] hydrogenase, and its associated cytochrome *b* subunit, were upregulated by 3.5-, 3.6-, and 2.9-fold respectively (to a maximum of 3105 FPKM) in response to O_2_ limitation (Figures 2A,C). Likewise, genes associated with hydrogenase maturation, nickel incorporation and nickel transport were also significantly upregulated (Supplementary Table S1). In contrast, the cytosolic Group 3b [NiFe] hydrogenase was constitutively transcribed at low levels (FPKM < 125).

We further analyzed RNA-seq data to investigate the influence of nitrogen limitation on transcriptional regulation within *Methylacidiphilum* sp. RTK17.1. During chemostat cultivation in the absence of a supplied nitrogen source (NH_4_^+^), 66 genes were upregulated (*p-value*: < 0.001; FDR: < 0.02) and 13 genes were downregulated (*p-value*: < 0.001; FDR: < 0.05) when compared to nitrogen excess growth conditions. Consistent with the onset of N_2_ fixation, a seven-gene operon encoding nitrogenase structural subunits and cofactor biosynthesis proteins (*nifHDKENX*, Minf_1870–1876) was upregulated (average 6.4 Log_2_FC; Figures 2B,C). In addition, numerous other genes involved in nitrogenase transcriptional regulation (*nifA*, Minf_0464), cofactor biosynthesis (*nifB*, Minf_0453), stabilization (*nifW*, Minf_0472), and nitrate (Minf_1096) and NH_4_^+^ transport (Minf_1075) were also significantly upregulated (Supplementary Table S1). Hydrogenase expression was not significantly different between the conditions, being high for the Group 1d [NiFe]-hydrogenase and low for the Group 3b [NiFe]-hydrogenase.

## Discussion

The accumulation and storage of carbon and energy as polymeric reserves is a common strategy employed by microorganisms during unbalanced growth to fortify them against periods of environmental starvation (Wilson et al., 2010). Nitrogen limitation is often cited as triggering the accumulation of carbon-rich reserve polymers (Wanner and Egli, 1990), but there is a lack of detailed understanding of the underlying mechanisms responsible for their production. As with many heterotrophic species, methanotrophs often produce carbon-rich polymers; however despite the prevalence of glycogen production within the Gammaproteobacteria methanotrophs (Pieja et al., 2011a), research has primarily focused on the physiology of polyhydroxybutyrate storage in the Alphaproteobacteria methanotrophs (Pieja et al., 2011a, b; Sundstrom and Criddle, 2015). The requirement for organic carbon compounds to provide both the respiratory energy and carbon necessary for anabolic processes for methanotrophs makes it difficult to untangle the roles of nitrogen, carbon, and energy availability (e.g., ATP) in the production of intracellular glycogen. However, as verrucomicrobial methanotrophs fix CO_2_ for carbon and supplement their energy requirements via the oxidation of H_2_ (Mohammadi et al., 2016; Carere et al., 2017), this affords an opportunity (obfuscated by the Type I and II methanotrophs) to investigate the influence of nitrogen, carbon, and energy availability independently.

Our findings show that H_2_ oxidation influences the production of intracellular glycogen reservoirs in the thermoacidophilic methanotroph, *Methylacidiphilum* sp. RTK17.1. During chemostat experiments, the maximum glycogen content of *Methylacidiphilum* sp. RTK17.1 occurred within cells grown in the absence of H_2_, under nitrogen- and O_2_- limiting growth conditions [48.86 (± 4.32)%; Table 1 and Figure 1A]. With respect to other studies reporting on the production of carbon storage polymers in methanotroph species, the glycogen content values observed for *Methylacidiphilum* sp. RTK17.1 are generally congruent. In the closely related thermoacidophile, *Methylacidiphilum fumarolicum* SolV, a maximum glycogen content of 36% (w/w) was observed (Khadem et al., 2012a) in nitrogen-limited, batch grown cells and a similar value (33% w/w) has been reported in the halotolerant methanotroph *Methylotuvimicrobium alkaliphilum* 20Z [formerly *Methylomicrobium alkaliphilum* 20Z (Khmelenina et al., 1999; Orata et al., 2018)]. A maximum of 42.8 (± 17.5)% (w/w) glycogen has been reported in the industrially promising methanotroph, *Methylotuvimicrobium buryatense* 5GB1 [formerly *Methylomicrobium buryatense* 5GB1 (Orata et al., 2018)], during batch-growth on methanol, with up to 13.1 (± 4.0)% (w/w) glycogen reported during O_2_-limited chemostat growth on methane (Gilman et al., 2015).

The variability in reported glycogen content within methanotrophs (intraspecies) and between experimental trials (interspecies) is almost certainly a consequence of both underlying physiological characteristics and the inherent challenges associated with characterizing dynamic batch growth environments. We therefore sought to perform a series of carbon-excess (CO_2_ and CH_4_) steady-state experiments to gain insight into the mechanisms governing glycogen production in *Methylacidiphilum* sp. RTK17.1. With respect to O_2_ and nitrogen limitation, rates of growth (inferred from observed biomass productivity rates), and changes to glycogen, and to a lesser extent total protein and amino acid contents, were consistent with a cell’s expected response to unbalanced growth conditions. While previous studies have reported significant changes to the amino acid composition of *Staphylococcus aureus* cultures in response to variable environmental conditions (Alreshidi et al., 2015, 2016), we observed no change to the relative abundance of specific amino acid residues in *Methylacidiphilum* sp. RTK17.1 cultures under the conditions tested. Nevertheless, a 144% increase in glycogen content was observed during unbalanced growth following the removal of H_2_ gas supply. Depriving *Methylacidiphilum* sp. RTK17.1 cultures of the respiratory energy gains afforded from H_2_ gas oxidation is demonstrative of how this strain dynamically allocates carbon, nitrogen and energy resources.

The observation that *Methylacidiphilum* sp. RTK17.1 cells produce glycogen and grow more slowly in response to oxygen limitation is consistent with the occurrence of glycogen within the obligate chemolithoautotroph *Hydrogenovibrio marinus* when grown on H_2_ and CO_2_ under O_2_-limiting conditions (Nishihara et al., 1993). Similarly, production of polyhydroxybutyrate (PHB) has been reported within heterotrophically grown cultures of *Azotobacter beijerinckii* in response to oxygen limitation (Senior et al., 1972). We speculate that in the absence of sufficient oxygen, both glycogen and PHB reserves likely serve to not only store carbon and energy, but to maintain intracellular redox state. It is also plausible that the anabolic activities required for cell division (i.e., protein, DNA and RNA synthesis) were constrained by ATP availability under O_2_-limiting conditions. Given protein synthesis requires approximately 19 times more ATP (mmol ATP (g macromolecule)^–1^) than for saccharide polymerization (Stouthamer, 1979; Russell and Cook, 1995; Russell, 2007), even considering the ATP requirements of CO_2_ fixation, glycogen biosynthesis likely represents an energetic *‘cost’* savings for *Methylacidiphilum* sp. RTK171 compared to the ATP-demands of cell growth. These modest increases to intracellular glycogen content in response to O_2_ limitation are unlikely to negatively impact biomass yields (Y_ATP_; Russell and Cook, 1995) while also benefiting cell survivability during periods of starvation. The additional burden imposed by nitrogen limitation not only created unbalanced growth conditions with respect to carbon and nitrogen, but also increased the cell’s ATP requirement via the nitrogenase reaction (N_2_ + 8H^+^ + 16ATP → 2NH_3_ + H_2_ + 16ADP). Under these growth conditions, glycogen accounted for nearly half of *Methylacidiphilum* sp. RTK17.1 cell mass. Supplementing CH_4_ oxidation with an alternative source of respiratory energy (H_2_), however, was sufficient to offset the ATP burden imposed by N_2_ fixation and consequently the production of intracellular glycogen was reduced and growth rates increased.

An alternative explanation for our chemostat observations is that synthesis of glycogen during energy-limiting conditions serves as a strategy for ‘metabolic anticipation’. The combined conditions of low O_2_, nitrogen, and H_2_ availability are highly limiting for a cell and further resource deprivation is likely to trigger a transition from growth to persistence. Thus, disproportionately allocating biomass into storage compounds under this condition may serve as a ‘bet-hedging’ strategy to enable longer-term survival when conditions worsen. Indeed, the synthesis and storage of intracellular carbon polymers is commonly associated with an increase in viability during periods of environmental starvation. As with PHB, glycogen catabolism supplies reduced electron carriers (e.g., NADH) into the respiratory chain, thereby enabling the continuation of metabolic processes in the absence of an exogenous energy supply (e.g., CH_4_ or H_2_). A reduced lag phase following CH_4_ starvation has previously been linked to the catabolism of glycogen reservoirs within the methanotroph *M. fumarolicum* SolV (Khadem et al., 2012a). Likewise, in *Methylacidiphilum* sp. RTK17.1 cultures, we interpret depletions in cellular glycogen content throughout prolonged incubations at 4°C (in the absence of CH_4_) as evidence it was being consumed to promote survival (Supplementary Figure S1). Within oxic/anoxic habitats, it seems evident that *Methylacidiphilum* sp. RTK17.1 distributes carbon, energy and nitrogen resources during methanotrophic or mixotrophic growth to fulfill the metabolic demands imposed for cell persistence and/or proliferation (Hanson and Hanson, 1996). Similar phenomena of metabolic anticipation have been observed in other species, for example mycobacteria, which accumulate storage compounds such as triacylglycerols during the early hypoxic response (Daniel et al., 2004; Eoh et al., 2017).

While hydrogenase, methane monooxygenase and nitrogenase all displayed evidence of significant transcriptional regulation in response to O_2_ and nitrogen limitation, the genes associated with glycogen metabolism were constitutively expressed. These results are consistent with previous findings within the verrucomicrobial methanotrophs (Khadem et al., 2010; Khadem et al., 2012a; Mohammadi et al., 2016; Carere et al., 2017) and suggests the enzymes associated with glycogen metabolism may be allosterically regulated in response to high carbon (i.e., fructose 1,6-bisphosphate) and/or energy contents (i.e., ATP/AMP), as described in other bacterial species (Wilson et al., 2010). Consistent with other verrucomicrobial methanotrophs (Dunfield et al., 2007; Pol et al., 2007; Op den Camp et al., 2009), *Methylacidiphilum* sp. RTK17.1 also possesses three phylogenetically distinct *pmoCAB* operons. Based on observed ratios of non-synonymous versus synonymous substitution rates in *pmoA* orthologs, it has been proposed that the pMMOs encoded in *Methylacidiphilum spp*. serve functionally distinct roles (Op den Camp et al., 2009). The observation that *Methylacidiphilum* sp. RTK17.1 transcriptionally regulates pMMO expression in response to oxygen availability therefore supports this hypothesis and is congruent with reports of differential expression in response to oxygen limitation (Khadem et al., 2012a) and during growth on methanol (Erikstad et al., 2012). Finally, it is noteworthy to include that the transcriptional upregulation of the Group 1d [NiFe] hydrogenase occurred in response to O_2_ limitation; whereas nitrogenase upregulation was induced by nitrogen availability. The transcriptional decoupling of these two enzymes is further evidence that the physiological role of H_2_ oxidation in methanotrophs (Mohammadi et al., 2016; Carere et al., 2017) is distinct from recycling H_2_ produced during the nitrogen fixation reaction (Bont, 1976; Dixon, 1976; Chen and Yoch, 1987). Collectively, these findings indicate while H_2_ oxidation is sufficient to partially offset the energetic costs associated with N_2_ fixation, the regulation of this enzyme is transcriptionally uncoupled from nitrogen availability.

## Data Availability

The raw and processed transcriptome sequence files (accession numbers GSM3872525–GSM3872529) were deposited into the Gene Expression Omnibus (GEO; https://www.ncbi.nlm.nih.gov/geo/) for archival storage.

## Author Contributions

CRC, CG, and MS conceived the study. CRC, MS, CG, CC, DG, and BM contributed to the experimental design. CRC, BM, CC, and HP conducted the bioreactor and wet lab experiments. CRC and HP performed the glycogen analysis. CRC, MS, and CG performed the transcriptome experiments and analysis. CRC, CG, MS, and DG wrote the manuscript with input from BM, HP, and CC.

## Conflict of Interest Statement

The authors declare that the research was conducted in the absence of any commercial or financial relationships that could be construed as a potential conflict of interest.

## Footnotes

1 https://www.ncbi.nlm.nih.gov/geo/
