# Supplementary material for: Hydrogen Oxidation Influences Glycogen Accumulation in a Verrucomicrobial Methanotroph
Source: Front Microbiol. 2019 Aug 16;10:1873. doi: 10.3389/fmicb.2019.01873 (PMC6706786; doi:10.3389/fmicb.2019.01873)
Supplement: Supplementary file 1 [file Data_Sheet_1.pdf]

Title: Hydrogen Oxidation Influences Glycogen Accumulation in a Verrucomicrobial Methanotroph

Carlo R. Carere[1*], Ben McDonald[2], Hanna A. Peach[3], Chris Greening[4], Daniel J. Gapes[2], Christophe Collet[2], Matthew B. Stott[5]

Author affiliations:
[1] Department of Chemical and Process Engineering, University of Canterbury, Christchurch, 8041 New Zealand
[2] Scion, Te Papa Tipu Innovation Park, Rotorua, New Zealand
[3] Geomicrobiology Research Group, Department of Geothermal Sciences, GNS Science, Taupō, 3384, New Zealand
[4] School of Biological Sciences, Monash University, Clayton, VIC, 3800, Australia
[5] School of Biological Sciences, University of Canterbury, Christchurch, 8041, New Zealand
* Corresponding author (email: carlo.carere@canterbury.ac.nz)

Supplemental Materials

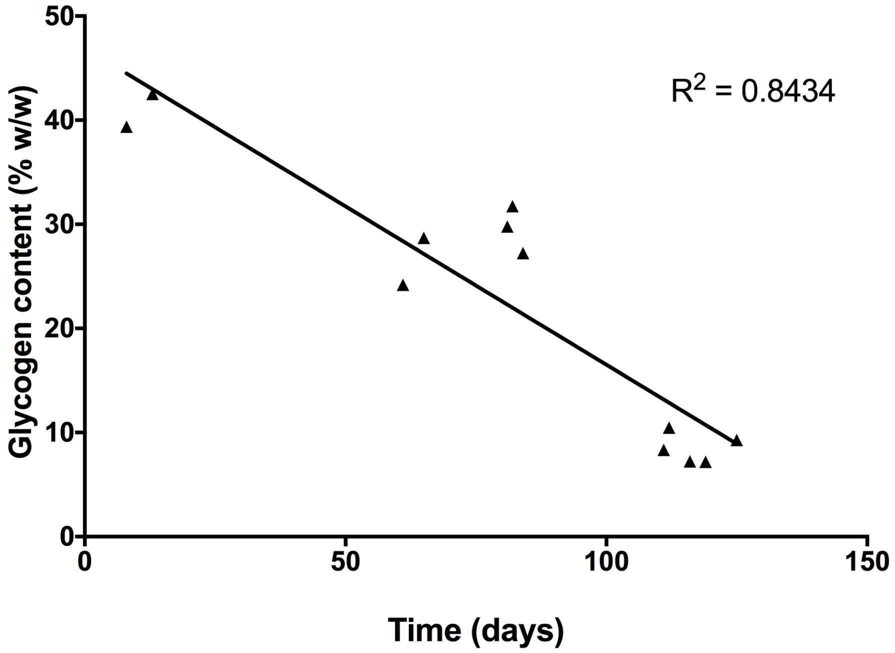

Figure S1. *Methylacidiphilum* sp. RTK17.1 intracellular glycogen reservoirs are slowly consumed during methane starvation at 4 °C. Multiple chemostat cultures samples (200 ml), grown under O_2-limited, N_2-fixing conditions in the absence of H_2 addition, were collected into sealed Pyrex 1l bottles (air headspace) and incubated at 4 °C without further nutrient supplementation. Periodically, the glycogen content of biomass was determined as described in the methods section.

Table S1. Expression profiles (FPKM) of key genes encoded in the *Methylacidiphilum* sp. RTK17.1 genome in response to changes to chemostat growth operation. Amino acid orthologs to *Methylacidiphilum infernorum* V4 are shown.

Gene	Predicted Function	Gene ID *	*M. infernorum* V4 ortholog			Expression (FPKM)				
			Accession	locus tag	% ID	N_2, low O_2, no H_2	N_2, low O_2, H_2	NH_4^+, low O_2, H_2	NH_4^+, O_2 replete, no H_2	NH_4^+, O_2 replete, H_2
**Methane oxidation to methanol**										
pmoC1	particulate methane monooxygenase, C subunit	KU509367	WP_012463847.1	Minf_1511	99	30039	37513	39558	21	20
pmoA1	particulate methane monooxygenase, A subunit	KU509368	ABX56601.1	Minf_1510	100	13819	17336	21307	34	19
pmoB1	particulate methane monooxygenase, B subunit		WP_012463845.1	Minf_1509	99	19965	24793	29076	39	39
pmoC2	particulate methane monooxygenase, C subunit	KU509373	WP_048810233.1	Minf_1500	99	618	550	722	4074	3951
pmoA2	particulate methane monooxygenase, A subunit	KU509371	WP_012463843.1	Minf_1507	99	4525	5583	6535	90972	67528
pmoB2	particulate methane monooxygenase, B subunit	KU509372	WP_012463842.1	Minf_1506	99	2623	3114	3891	50699	30091
pmoC2	particulate methane monooxygenase, C subunit	KU509370	WP_012463844.1	Minf_1508	99	2084	2928	3247	63702	45186
pmoC3	particulate methane monooxygenase, C subunit	KU509318	WP_012463927.1	Minf_1591	99	1	1	2	3	2
pmoA3	particulate methane monooxygenase, A subunit	KU509319	WP_012463926.1	Minf_1590	99	0	0	1	1	0
pmoB3	particulate methane monooxygenase, B subunit	KU509320	WP_012463925.1	Minf_1589	97	0	0	0	0	0
**Methanol oxidation to formate**										
xoxF	methanol dehydrogenase, large subunit	KU509410	WP_012463329.1	Minf_0992	99	9460	11221	14716	10834	9808
xoxJ	unknown function	Ga0079954	WP_012463332.1	Minf_0995	99	1045	1317	1279	1006	1028
mxaB	transcriptional regulator	KU509376	WP_012463785.1	Minf_1449	100	198	194	176	669	490
moxY	methanol utilization control sensor protein	KU509377	WP_012463784.1	Minf_1448	100	748	549	629	2874	1498
mxaR	ATPase involved in methanol oxidation	KU509402	WP_012463503.1	Minf_1166	97	7	6	9	7	8
**Formate oxidation to CO_2**										
fdsG	formate dehydrogenase, gamma subunit	KU509398	WP_048810187.1		97					
fdsB	formate dehydrogenase, beta subunit	KU509399	WP_012463566.1	Minf_1230	100	411	457	543	749	723
fdsA	formate dehydrogenase, alpha subunit	KU509400	WP_048810186.1	Minf_1229	99	741	763	731	1272	1096
fdsD	formate dehydrogenase delta subunit	KU509401	WP_048810185.1	Minf_1228	94	7	9	6	10	12

hpr	formate dehydrogenase	KU509384	WP_012463659.1	Minf_1323	99	658	680	968	635	889
**Hydrogen metabolism**										
hyaF	hydrogenase-1 operon protein	KU509388	WP_012463654.1	Minf_1318	99	382	487	561	54	61
hyaC	oxygen-tolerant membrane-bound [NiFe] - hydrogenase, cytochrome *b* subunit	KU509387	WP_012463655.1	Minf_1319	99	278	405	354	47	42
hyaB	oxygen-tolerant membrane-bound [NiFe] hydrogenase, large subunit	KU509386	WP_012463656.1	Minf_1320	99	2065	2767	2724	208	223
hyaA	oxygen-tolerant membrane-bound [NiFe] hydrogenase, small subunit	KU509385	WP_048810203.1	Minf_1321	99	2509	3105	3041	213	255
hyhB	NADP-coupled cytosolic bidirectional hydrogenase, FeS subunit	KU509477	WP_012464721.1	Minf_2387	99	206	245	195	158	147
crp	cyclic nucleotide-binding domain-containing protein	KU509476	WP_012464722.1	Minf_2388	97	91	93	61	62	51
hyhG	NADP-coupled cytosolic bidirectional hydrogenase, diaphorase subunit NAD(P)H-flavin reductase	KU509475	WP_012464723.1	Minf_2389	98	107	141	127	79	64
hyhS	NADP-coupled cytosolic bidirectional hydrogenase, small subunit	KU509474	WP_012464724.1	Minf_2390	98	106	124	118	67	87
hyhL	NADP-coupled cytosolic bidirectional hydrogenase, large subunit	KU509473	WP_012464725.1	Minf_2391	98	48	69	62	50	60
hypB	hydrogenase maturase protein, Ni storage	KU509355	WP_012464135.1	Minf_1799	99	1304	1068	1100	422	351
hypC	hydrogenase maturase protein, Fe- $(CN^-)_2$ -CO insertion	KU509357	WP_012464132.1	Minf_1796	100	1304	1359	1299	1460	1200
hypD	hydrogenase maturase protein, Fe- $(CN^-)_2$ -CO insertion	KU509358	WP_012464131.1	Minf_1795	99	318	346	329	291	382
hypE	hydrogenase maturase protein, CN^- ligand biosynthesis	KU509359	WP_012464130.1	Minf_1794	99	195	214	238	181	195
hypF	hydrogenase maturase protein, CN^- ligand biosynthesis	KU509356	WP_012464133.1	Minf_1797	99	79	93	103	47	57
**Copper homeostasis**										
cueR	copper resistance operon regulatory protein	KU509440	WP_012462789.1	Minf_0449	99	164	139	142	139	190
copA	Cu(I)-translocating P-type ATPase/ multicopper oxidase, copper-binding site	KU509375	gb|ABX56608.1|		99					
cueO	multicopper oxidase family protein	KU509374	WP_048810228.1	Minf_1469	99	1410	1628	1717	1130	922

cusA	copper efflux pump	KU509461	WP_012462437.1	Minf_0095	99	76	76	85	89	84
cusB	copper efflux pump membrane fusion protein	Ga0079954	WP_012462436.1	Minf_0094	98	106	106	121	104	121
cusS	copper sensor histidine kinase	KU509433	WP_012462941.1	Minf_0601	99	54	54	53	57	75
cusR	DNA binding copper response regulator	KU509432	WP_048810437.1	Minf_0602	99	258	367	307	249	318
**Coenzyme PQQ synthesis**										
pqqB	coenzyme PQQ synthesis protein B	KU509397	WP_012463570.1	Minf_1234	99	2186	2568	2367	2576	1491
pqqC	coenzyme PQQ synthesis protein C	KU509396	WP_012463571.1	Minf_1235	99	900	1110	971	697	547
pqqD	coenzyme PQQ synthesis protein D	KU509395	WP_048810188.1	Minf_1236	98	593	695	564	376	375
pqqD	coenzyme PQQ synthesis protein D	KU509434	WP_012462864.1	Minf_0524	97	16	16	13	16	13
pqqE	coenzyme PQQ synthesis protein E	KU509394	WP_048810189.1	Minf_1237	99	444	386	354	268	286
pqqA	Coenzyme PQQ synthesis protein A	KY820885	WP_012463569.1	Minf_1233	100	5251	5719	8763	12668	7397
No homologs of *pqqG pqqF*										
**Glycogen synthesis**										
mdoG	glycans biosynthesis protein	KU509364	WP_012463952.1	Minf_1616	98	32	37	42	21	17
mdoG	glycans biosynthesis protein	KU509365	WP_012463951.1	Minf_1615	99	103	97	83	58	72
	glucan elongation module (mdoH-like)	KU509366	WP_048810251.1	Minf_1612	99	46	50	55	50	36
GH57	glycosyl hydrolase family 57	KU509382	WP_012463665.1	Minf_1329	99	133	170	155	140	159
amyA	alpha amylase, catalytic domain	KU509383	WP_012463664.1	Minf_1328	98	244	267	254	268	229
glgA	glycogen synthase	KU509360	WP_012464092.1	Minf_1756	99	50	62	65	58	64
glgA	glycogen synthase (ADP-glucose)	KU509479	WP_048810560.1	Minf_2355	98	165	179	211	101	198
glgB	1,4-alpha-glucan branching enzyme	KU509409	WP_012463357.1	Minf_1020	99	123	134	122	93	146
glgC	glucose-1-phosphate adenylyltransferase	KU509460	WP_048810004.1	Minf_0180	99	235	216	241	222	174
glgP	starch phosphorylase	KU509451	WP_012462597.1	Minf_0255	99	305	325	337	274	384
glgP	starch phosphorylase	KU509431	WP_012462995.1	Minf_0658	99	462	470	450	311	349
glgP	starch phosphorylase	KU509429	WP_012463128.1	Minf_0791	99	140	178	195	171	236
gdb1	glycogen debranching enzyme (alpha-1,6-glucosidase)	KU509417	WP_012463227.1	Minf_0890	99	143	173	165	149	194
rfaG	glycosyltransferase	KU509418	WP_012463226.1	Minf_0889	99	18	18	19	17	20
glcD	glycolate oxidase	KU509414	WP_048810142.1	Minf_0895	98	198	238	273	264	281
glcD	glycolate oxidase FAD binding subunit	KU509413	WP_012463233.1	Minf_0896	98	22	22	27	34	20

aceB	malate synthase	KU509415	WP_012463229.1	Minf_0892	98	49	62	48	40	53
aceA	isocitrate lyase	KU509416	WP_048810457.1		98					
manB	phosphoglucomutase	KU509328	WP_012464463.1	Minf_2127	99	253	239	243	250	264
**Calvin-Benson Cycle**										
cbbS	ribulose 1,5-bisphosphate carboxylase, small subunit	KU509390	WP_012463599.1	Minf_1263	98	1957	1904	2444	1825	2219
cbbL	ribulose 1,5-bisphosphate carboxylase, large subunit	KU509389	WP_012463600.1	Minf_1264	99	2770	3154	3935	2894	3691
cbbX	probable Rubsico expression protein CbbX	KU509391	WP_012463598.1	Minf_1262	99	1044	1087	1183	1148	1167
pgk	phosphoglycerate kinase	KU509406	WP_048810158.1	Minf_1032	98	530	555	673	739	634
gapA	glyceraldehyde-3-phosphate dehydrogenase	KU509405	WP_012463370.1	Minf_1033	99	864	972	1345	1414	1408
fbaA	putative fructose-bisphosphate aldolase	KU509327	WP_048810542.1		98					
fbaA2	fructose-bisphosphate aldolase, class II	KU509448	WP_012462629.1	Minf_0287	100	1443	1397	1746	1136	1467
fbp	fructose-1,6-bisphosphatase I	KU509361	WP_012464020.1	Minf_1684	99	503	525	668	497	623
glpX	fructose-1,6-bisphosphatase II	KU509381	WP_012463695.1	Minf_1359	100	1115	1093	1080	1256	914
tktB	transketolase	KU509393	WP_012463596.1	Minf_1260	99	866	985	1020	957	1145
xfp	xylulose-5-phosphate/fructose-6-phosphate phosphoketolase	KU509412	WP_012463240.1	Minf_0903	99	255	262	264	233	298
tpiA	triosephosphate isomerase	KU509407	WP_012463368.1	Minf_1031	98	479	489	573	699	595
rpiB	ribose 5-phosphate isomerase B	KU509459	WP_012462539.1	Minf_0197	99	732	706	692	726	702
prkB	phosphoribulokinase	KU509392	WP_012463597.1	Minf_1261	100	848	903	1043	837	1115
rpe	ribulose-phosphate 3-epimerase	KU509354	WP_012464174.1	Minf_1838	99	353	391	477	254	394
**TCA cycle**										
gltA	citrate synthase	KU509447	WP_012462674.1	Minf_0334	99	641	528	509	363	608
acnA	aconitate hydratase	KU509408	WP_012463364.1	Minf_1027	99	190	163	130	121	295
icd	isocitrate dehydrogenase	KU509411	WP_012463276.1	Minf_0939	99	655	514	462	644	733
sucA	2-oxoglutarate dehydrogenase, E1 component	KU509464	WP_048810403.1	Minf_0067	98	703	497	333	458	772
sucB	2-oxoglutarate dehydrogenase, E2 component, dihydrolipoamide succinyltransferase	KU509463	WP_012462410.1	Minf_0068	98	420	412	451	400	622

lpdA	pyruvate/2-oxoglutarate dehydrogenase, E3 component, dihydrolipoamide dehydrogenase	KU509462	WP_012462411.1	Minf_0069	99	256	285	267	230	373
lpdA	pyruvate/2-oxoglutarate dehydrogenase, E3 component, dihydrolipoamide dehydrogenase	KU509362	WP_012463971.1	Minf_1635	98	382	351	320	287	335
sucD	succinyl-CoA synthetase subunit alpha	KU509403	WP_012463383.1	Minf_1046	99	347	382	324	254	612
sucC	succinyl-CoA synthetase subunit beta	KU509404	WP_048810162.1	Minf_1045	99	533	309	238	259	890
sdhB	succinate dehydrogenase, catalytic subunit	KU509442	WP_012462728.1	Minf_0388	99	380	305	305	253	358
sdhA	succinate dehydrogenase, flavoprotein subunit	KU509441	WP_012462729.1	Minf_0389	98	319	294	260	233	371
sdhC	succinate dehydrogenase, cytochrome b subunit	KU509443	WP_012462727.1	Minf_0387	98	268	179	138	191	147
fumC	fumarase	KU509428	WP_012463130.1	Minf_0793	100	254	331	335	273	410
mdh	malate dehydrogenase	KU509380	WP_048810212.1		99					
**ATP Synthase**										
atpD	F-type H^+-transporting ATPase subunit beta	KU509426	WP_048810122.1	Minf_0839	99	162	201	191	165	166
atpC	F-type H^+-transporting ATPase subunit epsilon	KU509425	WP_012463177.1	Minf_0840	99	145	145	142	137	128
	ATP synthase protein I	KU509424	WP_012463178.1	Minf_0841	98	68	57	51	61	49
atpB	ATP synthase F0 subcomplex A subunit	KU509423	WP_012463179.1	Minf_0842	98	75	76	72	60	70
atpE	F-type H^+-transporting ATPase subunit c	KU509422	WP_012463180.1	Minf_0843	98	86	96	97	60	63
atpF	ATP synthase F0 subcomplex B subunit	KU509421	WP_012463181.1	Minf_0844	99	382	341	421	322	280
atpA	F-type H+-transporting ATPase subunit alpha	KU509420	WP_012463182.1	Minf_0845	99	82	119	112	84	104
atpG	F-type H+-transporting ATPase subunit gamma	KU509419	WP_012463183.1	Minf_0846	100	101	123	94	90	103
atpH	F-type H^+-transporting ATPase subunit delta	KU509469	WP_012464754.1	Minf_2420	99	1661	2092	2473	1441	2144
atpB	F-type H^+-transporting ATPase subunit a	KU509472	WP_012464751.1	Minf_2417	99	1650	1964	2579	1681	1702
atpE	F-type H^+-transporting ATPase subunit c	KU509471	WP_012464752.1	Minf_2418	100	3536	3881	4151	3415	3505
atpF	F-type H^+-transporting ATPase subunit b	KU509470	WP_048810391.1	Minf_2419	99	4700	4389	5540	3788	4236
atpA	F-type H^+-transporting ATPase subunit alpha	KU509468	WP_012464755.1	Minf_2421	99	2501	2513	2846	1891	2890
atpG	F-type H^+-transporting ATPase subunit gamma	KU509467	WP_012464756.1	Minf_2422	99	1588	1364	1711	1314	1779
atpD	F-type H^+-transporting ATPase subunit beta	KU509466	WP_012464757.1	Minf_2423	100	1984	1906	2136	1731	2270
atpC	F-type H^+-transporting ATPase subunit epsilon	KU509465	WP_012464758.1	Minf_2424	100	3651	3035	3783	2972	2887

**NADH dehydrogenase**										
nuoB	NADH-quinone oxidoreductase subunit B	KU509458	WP_012462551.1	Minf_0209	99	922	1094	1013	613	1179
nuoA	NADH-quinone oxidoreductase subunit A	KU509480	WP_012464634.1	Minf_2300	99	900	716	759	966	694
nuoC	NADH-quinone oxidoreductase subunit C	KU509457	WP_012462552.1	Minf_0210	100	1410	1522	1849	1411	2286
nuoD	NADH-quinone oxidoreductase subunit D	KU509456	WP_012462553.1	Minf_0211	99	1020	1135	1213	922	1438
nuoE	NADH-quinone oxidoreductase subunit E	KU509455	WP_012462554.1	Minf_0212	99	1241	972	962	854	1327
nuoF	NADH-quinone oxidoreductase subunit F	KU509454	WP_012462555.1	Minf_0213	99	1064	982	1111	785	1455
nuoG	NADH-quinone oxidoreductase subunit G	KU509453	WP_012462556.1	Minf_0214	99	1397	1390	1354	1104	1719
nuoH	NADH-quinone oxidoreductase subunit H	KU509452	WP_012462557.1	Minf_0215	99	359	326	343	324	387
nuoI	NADH-quinone oxidoreductase subunit I	KU509321	WP_048810348.1	Minf_2216	99	174	217	249	228	276
nuoJ	NADH-quinone oxidoreductase subunit J	KU509322	WP_048810347.1	Minf_2215	99	892	978	1077	800	1142
nuoK	NADH-quinone oxidoreductase subunit K	KU509323	WP_012464548.1	Minf_2214	100	190	288	248	142	276
nuoL	NADH dehydrogenase subunit L	KU509324	WP_012464547.1	Minf_2213	98	426	497	506	334	583
nuoM	NADH-quinone oxidoreductase subunit M	KU509325	WP_012464546.1	Minf_2212	99	297	281	353	272	466
nuoN	NADH dehydrogenase subunit N	KU509326	WP_012464545.1	Minf_2211	99	373	399	497	358	747
**Cytochrome C oxidase and Complex III (ACIII)**										
cyoE	protoheme IX farnesyltransferase	KU509378	WP_012463763.1	Minf_1427	99	260	248	273	59	59
ctaA	cytochrome c oxidase assembly protein subunit 15	KU509379	WP_012463762.1	Minf_1426	99	704	601	648	182	129
cyoC	cytochrome c oxidase subunit 3	KU509342	WP_012464286.1	Minf_1950	99	1031	1216	1177	498	620
cyoA	cytochrome c oxidase subunit 2	KU509344	WP_012464280.1	Minf_1944	100	2021	2139	2021	1162	1433
cyoB	cytochrome c oxidase subunit 1	KU509341	WP_012464287.1	Minf_1951	99	1404	1406	1579	974	1301
	caa$_3$-type oxidase, subunit IV	KU509343	WP_048810305.1	Minf_1949	98	1549	1307	1348	570	695
cccA	cytochrome c oxidase, *cbb*$_3$-type, subunit III	KU509340	WP_012464288.1	Minf_1952	100	390	393	436	377	468
cytC	cytochrome c	KU509481	WP_012463333.1	Minf_0996	97	462	568	606	480	590
ccoO	cytochrome c oxidase *cbb*$_3$-type subunit 2	KU509339	WP_012464289.1	Minf_1953	99	216	257	263	163	300
ccoN	cytochrome c oxidase *cbb*$_3$-type subunit 1	KU509338	WP_048810306.1	Minf_1954	99	188	184	202	192	198
	cytochrome c & quinol oxidase polypeptide I	KU509482	WP_012462607.1	Minf_0265	91	4594	5747	5636	66	48
	heme/copper-type cytochrome/quinol oxidase, subunit 1	KU509483	WP_012462607.1	Minf_0265	99					

ActG†	Hypothetical membrane-associated protein	Ga0079954	WP_012464292.1	Minf_1956	99	1466	1080	1525	1322	1202
ActF†	quinol:cytochrome c oxidoreductase quinone-binding subunit 2	KU509337	WP_012464293.1	Minf_1957	99	158	135	165	160	206
cccA – *ActE*†	cytochrome c oxidase, cbb_3-type, subunit III	KU509336	WP_012464294.1	Minf_1958	99	203	237	274	198	258
ActD†	Hypothetical membrane-associated protein	Ga0079954	WP_012464295.1	Minf_1959	100	279	251	332	210	292
ActC†	prokaryotic molybdopterin-containing oxidoreductase family, membrane subunit	KU509335	WP_012464296.1	Minf_1960	99	166	180	189	146	250
hybA – *ActB*†	prokaryotic molybdopterin-containing oxidoreductase family, iron-sulfur binding subunit	KU509334	WP_012464297.1	Minf_1961	99	341	359	388	273	518
ActA†	cytochrome c7	KU509333	WP_012464298.1	Minf_1962	99	508	448	518	445	700
**Nitrogen fixation**										
nifH	Mo-nitrogenase iron protein subunit NifH	KU509347	WP_012464212.1	Minf_1876	99	15021	16403	37	50	54
nifV	homocitrate synthase	KU509346	WP_012464213.1	Minf_1877	99	652	476	19	18	33
nifD	Mo-nitrogenase MoFe protein subunit NifD precursor	KU509348	WP_012464210.1	Minf_1874	99	7795	8547	33	47	40
nifK	Mo-nitrogenase MoFe protein subunit NifK	KU509349	WP_048810527.1	Minf_1873	99	2578	2772	20	28	24
nifE	nitrogenase molybdenum-cofactor synthesis protein	KU509350	WP_012464208.1	Minf_1872	99	1546	1642	36	34	41
nifN	nitrogenase molybdenum-iron protein NifN	KU509351	WP_048810526.1	Minf_1871	99	996	1100	33	26	60
nifX	nitrogen fixation protein NifX	KU509352	WP_012464206.1	Minf_1870	100	997	1088	23	27	61
